# Effects of Gelatin/Chitosan and Chitosan Active Films with Rice Bran Extract for the Preservation of Fresh Pork Meat

**DOI:** 10.3390/gels11050338

**Published:** 2025-04-30

**Authors:** María Cabeza de Vaca, Rosario Ramírez, Javier Rocha-Pimienta, David Tejerina, Jonathan Delgado-Adámez

**Affiliations:** Technological Institute of Food and Agriculture (INTAEX), Center for Scientific and Technological Research of Extremadura (CICYTEX), Avda, Adolfo Suárez s/n, 06007 Badajoz, Spain; mariarosario.ramirez@juntaex.es (R.R.); javier.rocha@juntaex.es (J.R.-P.); david.tejerina@juntaex.es (D.T.)

**Keywords:** active film, gelatin, chitosan, active biodegradable packaging, meat preservation, rice bran extract (RBE)

## Abstract

Films formulated with gelatin and chitosan (GL/CH) or chitosan (CH), without or with 0.3% and 0.5% concentrations of rice bran extract (RBE), have been developed. The migrations of rice bran extract and the antioxidant and antimicrobial properties in vitro have been assessed. The effects of the film formulations in maintaining color stability, oxidative status and microbial loads on fresh pork meat during 9 days of refrigerated storage were studied. For the films, releases of γ-oryzanol only were observed in low polarity simulant. The highest migrations and antioxidant activity were related to gelatine films, enhanced with the addition of rice bran extract. Only chitosan films showed antimicrobial activity in vitro against *Escherichia coli* and *Listeria innocua*, reaching decreases of 7.68 and 8.06 Log CFU at 72 h, respectively. Both gelatin/chitosan and chitosan films prevented the color changes in meat during storage, preventing the paleness, and chitosan films also provoked an increment of redness until 2.88 units of CIE b* at day 9. The films did not prevent either lipid or protein oxidation in meat, despite the rice bran extract inclusion, even increasing the lipid oxidations with chitosan films. However, all films helped to control the microbial counts in meat throughout all the storage, with chitosan being the most effective films, especially with the addition of RBE. Overall, gelatin/chitosan and chitosan films offer a sustainable alternative for fresh pork meat packaging.

## 1. Introduction

Spoilage provoked by microorganisms and physicochemical oxidations is the principal cause of damage to fresh meat products. To reduce the microorganisms and lipid oxidations, protection of the meat with plastic films as inert barriers to gas, microbes and water vapor is one of the main strategies to increase the meat shelf-life. The active films, with bioactive compounds, are widely applied to improve their preservative properties due to the migration of the active agents into the headspace or onto the food surface [1,2,3]. On the other hand, due to a growing social awareness of environmental and economic sustainability, new European policies have aimed to reduce the use of non-biodegradable plastics derived from fossil fuels and promote their replacement by biodegradable materials. Thus, the development of biodegradable biopolymers, derived from biological sources, represents an eco-friendly alternative to conventional films for the food packaging industry [4,5].

In this context, reutilization and economic valorization of agro-industrial by-products, with the development of new value-added functions, is a suitable alternative to promote economic sustainability. Among these agro-industrial by-products, chitosan is a natural polymer derived from chitin, the second most plentiful natural polysaccharide present in fungi, animals and microorganisms [6], and gelatin is a noteworthy protein-based biopolymer derived from mammals and fish [7]. Both compounds represent two edible, abundant and cheap biomaterials easily obtainable on an industrial scale from agro-industrial wastes [7,8,9] and feasible to use as biodegradable films for the packaging of different food products, being a suitable alternative to non-degradable plastic for single-use packaging due to their natural origin, high biodegradability and suitable obtention [10].

However, to improve their technical and physical properties, gelatin and chitosan-based films require plasticizers or additives that can release toxic compounds during the biopolymer degradation. For this reason, the substitution of chemical additives by active natural additives in biopolymer films is particularly interesting to eliminate their potential toxicity and enhance their antioxidant and antimicrobial preservative properties [11]. In this sense, gelatin and chitosan-based active films, with active properties like antioxidant or antimicrobial activity, formulated with several essential oils and natural extracts, have been previously used in the preservation of meat products, and their efficacy against microbiological growth and oxidant processes extensively tested [12,13,14,15,16,17], though both biopolymers have antioxidant and antimicrobial properties per se [18]. Among the sources of active compounds with antimicrobial and antioxidant properties, we can find the rice bran extract (RBE) [19], that constitutes a plentiful and inexpensive agro-industrial by-product, suitable for meat preservation [20]. Specifically, the main bioactivity of RBE is due to a mix of natural lipophilic active compounds like tocopherols and γ-oryzanol, with γ-oryzanol being the main and most active compound linked rice bran extract. The γ-oryzanol is a mixture of ferulate esters of triterpene alcohol, where the major components are cycloartenyl ferulate, 24-methylene cycloartenyl ferulate, campestanyl ferulate, and sitosteryl ferulate [21].

With all, the effect of adding RBE to active packages has been recently explored, improving on slice dry-cured Iberian ham the control of microorganism growth, delaying the oxidations and avoiding the meat discoloration during storage [22]. However, the inclusion of RBE in biobased PHA/PBH active films showed an innocuous effect on loin pork fresh meat [13]. Considering these results, the active biobased films formulated with gelatin, chitosan and RBE could be an eco-friendly, edible, non-toxic, low-cost and sustainable alternative to conventional plastic films for fresh meat preservation. Nonetheless, variations in film formulations can modify their physicochemical features and, subsequently, their protective properties [16,18,23,24,25]. For this reason, a deep evaluation of the final effects of the different formulations would be necessary, both in vitro and on meat products. Therefore, the present study aimed to evaluate the behavior of active biofilms based on gelatin and chitosan biopolymers formulated with RBE and their effectivity for preserving refrigerated fresh pork from the points of view of color preservation, oxidative prevention and microbiological control.

## 2. Results and Discussion

### 2.1. Films Characteristics

The RBE ethanolic extracts, obtained from solid liquid extraction with ethanol 100% from rice bran, presented a yield of 16.3 mg γ-oryzanol g^−1^ extract, an antioxidant activity of 13.5 mg Trolox equivalent g^−1^ extract, and a minimum inhibitory concentration (MIC) of 6.62% against *E. coli* and 6.42% against *L. innocua*, which was in line with the range of previous studies [19].

No RBE migration was detected in stimulant D1 (ethanol 50% solution), despite the amphipathic properties of γ-oryzanol compounds, with a phenolic hydroxyl group (-OH) and a long hydrophobic tail, that give those compounds a not strictly non-polar nature [26]. For simulant D2 (ethanol 95%), the release trends depended on the film and the RBE dose (Table 1). Thus, migrations were significantly higher for GL/CH film than for CH. Regarding the effect of RBE content, a significant effect (*p* ≤ 0.01) only was observed at 144 and 240 h, with some differences between films. For GL/CH, higher levels of γ-oryzanol in L-RBE than in H-RBE were observed (*p* ≤ 0.05). For GL/CH H-RBE the highest migrations were linked (*p* ≤ 0.05) from 48 h, and GL/CH L-RBE at 6, 72, 144 and 240 h. Regarding the evolution of migrations, variations on the γ-oryzanol releases took place throughout the whole assay for all films, except for CH L-RBE, which remained similar (*p* > 0.05) for the 240 h.

The maxima migrations were reached at 6 h in GL/CH-L-RBE, and at 48 h in GL/CH-H-RBE and CH-H-RBE (*p* ≤ 0.01). According to the literature, the release of active compounds from gelatin and chitosan films depends on the biopolymer, the active additive and the assay conditions. The polarities of the food simulant and the active compounds, as well as the temperature of the assay, are the most critical factors on compound releases, being especially low for temperatures under 40 °C [27,28] and for simulants with ethanol concentrations under 95% [1]. Therefore, the low polarity of γ-oryzanol from RBE, together with the low temperature of the present assay (30 °C), could explain the lack of detection of this compound for simulant D1. In the same way, Cabeza de Vaca et al. [13] for RBE added to PLA/PHB films, also detected migrations of RBE only in ethanol 95% and not in ethanol 50%, and [2] for chitosan films observed that migrations in fatty food simulant only occurred for essential oils but not for hydroalcoholic extracts with high polarity. With all, higher assay temperatures could increase the release of RBE compounds in the D1 for GL/CH and CH films until they are detectable. Regarding γ-oryzanol migrations in D2, the release trends of both films were in concordance with the previous bibliography. Thus, in general, the maxima velocity of active compound migrations was observed in the first 12–24 h reaching the equilibrium from 48–96 h for CH, GL/CH or PLA films [13,27,28,29]. On the one hand, the dissolving capacity, permeability, chemical structure or morphology of the biopolymer can facilitate or hinder the liberation of active molecules from films. In the case of the CH films, all these characteristics are closely related to biopolymer formulation. Specifically, a higher content of gelatin in GL/CH films decreased the moisture absorption capacity and the molecular mobility, increasing the solubility of films and the migrations [8,30,31]. These changes could explain, in part, the major liberation of γ-oryzanol linked to GL/CH films. The ability to release the additives is closely related to the type and hydrophobicity of the extracts [16,30]. However, the inclusion of active compounds also can alter the physicochemical properties of chitosan and gelatin films and affect the migrations [17]. In particular, the incorporation of rice bran oil can drive changes in morphologic and mechanical properties in films, like the case of PLA [32], which, with the final formulation, could explain the differential trend in γ-oryzanol releases from GL/CH and CH films.

Regarding the total antioxidant activity (TAA) in vitro (Table 2), and in concordance with Tessaro et al. [30], the GL/CH formulations showed higher values than CH in the whole assay (*p* ≤ 0.001).

In line with previous studies [18], GL/CH and CH films without RBE presented antioxidant activity per se, and the RBE addition only affected TAA at 2 and 6 h (*p* ≤ 0.05). But the effect of RBE in TAA depended on the film. Thus, whereas for GL/CH films the addition of RBE only enhanced the TAA for H-RBE (*p* ≤ 0.001) at 6 and 24 h, for CH films the addition of RBE significantly decreased the TAA (*p* ≤ 0.05) for the whole assay, but especially at 6 h for L-RBE. According to the bibliography, the application of essential oils and active extracts usually increases the antioxidant activity of chitosan or gelatin films [18,24,31], although it is not always significantly proportional to extract added [23,33], and even, in some cases, not being enhanced for some extracts doses [34]. Furthermore, in agreement with our findings, Tessaro et al. [30] found that the inclusion of *Eugenia uniflora* L. leaf hydroethanolic extract did not improve the antioxidant activity of chitosan films, whereas for films formulated with gelatin and chitosan the TAA increased. Although other factors may influence antioxidant activity, such as the uniform dispersion of rice bran extract in the film, Tessaro et al. [30] found no differences in the microstructure of CH, GL, and GL/CH films when *Eugenia uniflora* L. hydroethanolic extract oils were added. Various other factors, including the interactions between the compounds in the matrix, could explain the differences in antioxidant activity observed in the studied active films. With all, the higher proportion of active compounds in GL/CH or CH films does not always imply the higher release of active compounds nor an increment of antioxidant properties, as was observed in the present assay for RBE. Concerning the antioxidant stability for 24 h, an increase of TAA was observed only in GL/CH with no RBE and H-RBE and CH L-RBE treatments (*p* ≤ 0.05), keeping the rest of formulations similar values (*p* > 0.05) throughout the 24 h.

The antimicrobial activity of each film was determined as the inhibitory capacity against *Escherichia coli* and *Listeria innocua* (Table 3).

For GL/CH films no antimicrobial activity was detected. In contrast, all CH films were effective against *E. coli* and *L. innocua.* Against *E. coli,* the CH films presented antibacterial properties per se, with reductions of 5.93, 4.87 and 4.09 units in no-RBE respect the control at 24, 48 and 72 h, respectively. The addition of RBE improved the antimicrobial activity of CH films for *E. coli* from 48 h, with the highest inhibitory activity for H-RBE (*p* ≤ 0.001), decreasing the *E. coli* loads in 6.80 units at 48 h and 7.68 units at 72 h respect to the control batch. For *L. innocua* similar trends were observed, being all CH films effective throughout the 72 h. The no-RBE formulation decreased the loads, concerning the control, between 3.55 and 6,88 units. The addition of RBE enhanced the inhibitory of CH film, making it more effective with the H-RBE at 24 and 48 h. At 72 h similar effect was observed for both doses, decreasing the *L. innocua* loads with respect to control in 7.74 and 8.06 units for L-RBE and H-RBE, respectively. The bactericide and fungicide activity of chitosan biopolymers has been previously reported [35,36]. This antimicrobial potential of chitosan is due to several chemical and biological mechanisms at a cellular level that contribute to the final cell death, such as changes in the cell membrane permeability, the chelation of nutrients crucial to bacteria metabolism, the inhibition of gene expression, or the generation of reactive oxygen species (ROS) in bacterial cells [10]. However, the final antimicrobial activity depends on several factors like the biological source [6], the final molecular weight of chitosan biopolymer, showing a differential antibacterial potential against *E. coli* the chitosan with low and high molecular weight [37] due to the capacity to penetrate the cell surface [38], or the deacetylated degree of the NH2 groups, which exert the antimicrobial activity modifying the DNA/RNA and the protein synthesis [38]. For chitosan films, the antimicrobial properties can be modified by the acid solvent used in the chitosan film formation [6], given that their presence in the final films with a concentration over 200 ppm can show antibacterial activity [37], and they can affect the final pH of films and subsequently the presence of functionally active -NH^3+^ groups of chitosan responsible for the antimicrobial activity [38].

On the other hand, pure gelatin films showed no antimicrobial effect [8,35] or a slight inhibitory effect [7]. The incorporation of chitosan can develop the antimicrobial activity of gelatin films [8], but it depends on the chitosan final concentration [7]. In concordance with the present study, Gómez-Estaca et al. [39] neither detected an antimicrobial inhibition for the films that combined gelatin/chitosan in the same proportion as ours (3:1, gelatin: chitosan). With all, in the present assay, the lack of antimicrobial properties of GL/CH films can be explained by a low antimicrobial potential of the original gelatin and chitosan biopolymers or by an insufficient proportion of chitosan in the final formulation. On the other hand, though the addition of active extracts normally enhances the antimicrobial properties of chitosan or gelatin films [23,35,39], it depends on the active compounds and the microorganisms evaluated. In this sense, the incorporation of catechin did not result effective in gelatin pure films against *Pseudomonas aeruginosa*, *Escherichia coli*, *Bacillus cereus* and *Staphylococcus aureus* [31], and, according to Zemljič et al. [40], the addition of extracts of *Rubus fruticuns* leaves and *Juniperus oxycedrus* decreased antimicrobial inhibitory effect of pure chitosan coating. Moreover, in line with our results, the addition of black rice bran anthocyanin improved the antimicrobial activity of chitosan films only inside a dilution interval, showing the maxima efficacy for the medium concentrations [16]. With all, for gelatin and chitosan-based films the addition of extracts with antimicrobial activity does not always imply a proportional enhancement of their antimicrobial properties, like in the present study, in which the higher RBE dose did not result in a higher effectiveness of films.

### 2.2. Effect of Films with Rice Bran Extract for Fresh Pork Preservation

In the fresh pork loin, the global effects of the type of film, the RBE dose and the time of storage on the parameters analysed, as well as their interactions, are shown in Table 4.

Thus, the type of biopolymer utilized affected all studied colour parameters (*p* ≤ 0.01), as well as the lipid and protein oxidations and all studied microorganisms (*p* ≤ 0.05). The addition of RBE to the films resulted in a significant factor for CIE b*, chroma, hue, ΔE_storage_, mesophiles and total coliform contents (*p* ≤ 0.005). Overall, storage time showed a significant effect (*p* ≤ 0.05) on the evolution of almost all the studied parameters, but CIE a*, hue and *E. coli* loads. Regarding the interaction between the factors, the RBE content showed a crossing effect (*p* ≤ 0.05) with the type of film for CIE b*, chroma, hue, ΔE_initial_control_, ΔE_storage_, lipid oxidation, mesophiles, moulds and yeasts and total coliforms. A crossing effect was also observed between film type and storage time (*p* ≤ 0.05) for CIE L*, ΔE_initial_control_, ΔE_storage_, lipid oxidation and the counts of all studied microorganisms but *S. aureus*. The RBE addition showed an interaction with time factor (*p* ≤ 0.05) for the CIE b*, chroma, ΔE control T0, lipid oxidation, moulds and yeasts and total coliforms. Finally, a combined interaction between the three factors was observed for CIE L*, CIE b*, protein oxidations, mesophiles, moulds and yeast and total coliforms.

#### 2.2.1. Instrumental Color in Pork Loin

For the instrumental colour of fresh pork meat, the specific results for each color parameter are shown in Table 5.

In terms of CIE L, and similarly to Cabeza de Vaca et al. [13], the control samples increased the CIE L during storage, which is associated with the physiological changes during ageing. Those changes in luminosity are linked to fibre elongation, provoked by the pH and water holding variations during meat ageing, that modify the refraction of light through the myofibrils and the surface tissues, leading to the paleness of pork meat [41]. At day 1, the use of the films did not affect lightness. At day 5, only CH with L-RBE kept lower lightness than control (*p* ≤ 0.05). However, and unlike the results shown by T. Liu and Liu [42]. and Antoniewski et al. [4] for refrigerated pork meat with chitosan films, at day 9, except CH no-RBE and H-RBE, all films contributed to reducing the CIE L with respect to control (*p* ≤ 0.05). Because lightness increments are generally perceived by consumers as an increase of meat paleness, the reduction in CIE L observed with GL/CH and CH films at day 9 should be considered positively. Overall, the addition of RBE did not prove effective to preserve the lightness. For redness, in accordance with the literature [13,43], control samples trended to decrease CIE a* throughout storage, due to the browning discolouration of myoglobin in its oxidised forms [44]. On the other hand, whereas GL/CH films did not affect redness (*p* > 0.05), the effect of CH was variable. Thus, at day 1, CH increased (*p* ≤ 0.01) the redness, similarly with all the formulations, whereas at day 5, the CH film did not affect the CIE a* (*p* > 0.05). At day 9, the CH films increased the redness (*p* ≤ 0.001), reaching CIE a* values above 4.00 for the three formulations. With all, CH implied in all the assay higher redness than GL/CH (*p* ≤ 0.05), with CIE a* values for GL/CH films that did not exceed 2.74 and for CH over 3.76. In refrigerated pork meat, increments of redness with chitosan films were also observed by T. Liu and Liu [42] and no effect of gelatine on redness was shown by Antoniewski et al. [4]. This effect of enhancement of CIE a* of meat products linked to Chitosan films can be mainly due to their ability to reduce oxidation processes in myoglobin proteins. This antioxidant property of chitosan, attributed to its ability to chelate free iron released during myoglobin degradation, plays an important role in this effect [45]. By chelating free iron, chitosan inhibits the catalysis of oxidative reactions that typically lead to the formation of metmyoglobin, which imparts a brownish color and reduces redness. Consequently, by maintaining myoglobin in its reduced form, the desired bright red color of fresh meat is maintained, as reflected by an increase in CIE a* values. Thus, according to the above literature, higher redness linked to CH (2% chitosan) than for GL/CH films (1% chitosan and 3% gelatin) in the present study was expected. Finally, the addition of RBE to films, similarly to Cabeza de Vaca et al. [13], did not cause any variation on CIE a*, despite the protective effect on redness reported for RBE by Martillanes, Ramírez, et al. [20] in fresh pork burgers.

The CIE b* values of the control samples slightly increased the yellowness during storage, with values ranging in 9.12–9.15 at day 1 and 9.30–9.89 at day 9. In general, and according to T. Liu and Liu [42], for refrigerated pork meat, the application of GL/CH and CH films helped to decrease the yellowness, except CH at day 1, though depending on the formulations. At day 1, only GL/CH with no RBE showed lower CIE b* than control (*p* ≤ 0.05), and at days 5 and 9, GL/CH with the three formulations and CH with L-RBE significantly decreased the CIE b* (*p* ≤ 0.01). Thus, both films helped to control the CIE b* variations at the end of the assay. On the other hand, and according to Martillanes, Ramírez, et al. [20], the addition of RBE barely affected yellowness, decreasing the CIE b* only for L-RBE dose for CH at day 5, not showing any effect for the rest of the batches (*p* > 0.05).

Regarding the polar coordinates, the chroma and hue of loins are shown in the Supplementary Material. Our results showed that control samples tended to maintain the chroma or saturation, and to increase the hue values toward brown discolorations, being in concordance with the previous literature [13,20]. These color modifications can be mainly explained by the oxidation process of myoglobin, common in the ageing process of pork meat leading to browning discoloration and yellowness linked to oxidized forms, at the expense of the redness-reduced forms [44]. Considering the effects of films, at day 1, only GL/CH no-RBE significantly (*p* ≤ 0.05) decreased the chroma. At day 5, all the GL/CH and CH formulations showed lower chroma than the control (*p* ≤ 0.05) but CH with H-RBE. At the end of storage, all GL/CH decreased the color saturation (*p* ≤ 0.001) whereas CH did not affect it. Because of the predominant yellow tone of meat samples, with CIE b* values higher than CIE a*, in general, the variations of chroma showed a similar trend as CIE b*, with the lowest saturations linked to GL/CH, being significantly lower than CH films in all no-RBE batches, L-RBE at day 1 and H-RBE at day 9. The hue, or color tone, was not affected by GL/CH films throughout the whole assay, and CH kept tones redder than control in all batches, but L-RBE at day 1 and no-RBE at day 5, due to the highest CIE a* values linked to CH batches. In order to know the global effect of the films on meat color, the color changes with respect to initial meat samples without treatment (control at day 1) were calculated (∆E_initial_control)_. In general, both biopolymers showed similar behavior and the RBE dose did not affect the ∆E_initial_control_. Moreover, an interaction between film and storage time was observed (Table 4). According to NBS (National Bureau of Standards) the total color variations (∆E) based on visual perception ranges from not noticeable (0–0.5), slightly noticeable (0.5–1.5), noticeable (1.5–3.0), well-visible (3.0–6.0), great difference (6.0–12.0), to very great difference (>12). Thus, the application of both films involved “noticeable color changes” at day 1, not finding differences between GL/CH vs. CH (*p* > 0.05). At day 5, ∆E_initial_control_ was not affected by the application of films (*p* > 0.05), and no significant differences were found between GL/CH and CH films, but for L-RBE dose, despite color changes of control and CH could be considered “well visible” and for GL/CH as “noticeable”. At day 9, GL/CH helped to maintain the color variations with values smaller than 3.00 (“noticeable”), even showing values of ∆E_initial_control_ lower than control (*p* ≤ 0.05) in no RBE and L-RBE treatments. In contrast, the application of CH did not improve the color variations at day 9 (*p* > 0.05). The addition of RBE did not improve the color changes for GL/CH and CH films in the whole assay. On the other hand, the evolution of color changes within each type of film respecting day 1 (ΔE_storage_) was similar for both GL/CH and CH biopolymers. For GL/CH and CH films, and similarly to Cardoso et al. (2019) [46], ΔE_storage_ trended to be lower than control but not significantly (*p* > 0.05). The addition of RBE did not affect ΔE_storage_, in the same way that was observed by Cabeza de Vaca et al. [13] for PLA/PHB active films with RBE for fresh pork meat and by H. W. Kim et al. [15] for natural antioxidants incorporated in chitosan coats. Oppositely, the addition of RBE in conventional packaging prevented the discoloration of Iberian sliced dry-cured ham during long storage [22] and the direct application of RBE in mince pork burgers improved the color preservation [20]. These positive effects can be due to the way of application of the RBE, since Martillanes et al. [22] spread the RBE in the film surface, instead of including it, with a final dose higher (0.3 g per film) than in our assay, whereas Martillanes, Ramírez, et al. [20] applied the RBE directly in the minced meat. Additionally, the lipophilic character of RBE would explain the effectiveness of the RBE active found by Martillanes et al. [22], due to the high-fat content of Iberian dry-cured ham compared to the fresh commercial pork loin used in the current assay.

#### 2.2.2. Lipid and Protein Oxidation in Pork Loin

The type of film and the time storage significantly affected lipid and protein oxidation in pork loin (Table 4). The GL/CH films showed no effect against TBA-RS lipid oxidation (Table 6), with similar values to control in the entire assay for all formulations. The CH films significantly increased the lipid oxidations when applied without RBE, especially at days 5 and 9 (*p* ≤ 0.001). Though the addition of RBE did not present a significant overall effect, an interaction between film type and RBE dose was observed (*p* ≤ 0.01) (Table 4). Thus, whereas for GL/CH the addition of RBE did not affect the TBA-RS (*p* > 0.05), for CH the RBE inclusion helped to control the increment of lipid oxidations linked to chitosan films at day 9, decreasing the TBA-RS values in L-RBE and H-RBE batches with respect to no-RBE (*p* ≤ 0.001). In contrast with the antioxidant activity observed in vitro for GL/CH and CH films (Table 2), significantly higher in GL/CH, a slight or no positive effect of films was observed for oxidation meat prevention. This discordance could be explained, in part, by the time and temperature conditions of both assays. Thus, the antioxidant activity of films was measured until 24 h, and the analysis of oxidations in meat started after 24 h of refrigeration, being a lack of information for the assay in vitro throughout the storage assay. Moreover, the antioxidant assay of films was carried out at room temperature (20–22 °C), whereas the meat storage assay was in refrigeration (4 °C), so the antioxidant properties and dynamics linked to the films could be modified at low temperatures [27,28].

Therefore, the addition of antioxidants in chitosan films to prevent lipid oxidations in meat can result in interest. In relation to the preservation of pork meat against protein oxidation (Table 6), the application of GL/CH films showed no effect, and CH helped to control the protein oxidations only for no-RBE at days 5 and 9 (*p* ≤ 0.05). Therefore, only no-RBE CH films could present some protective effect against protein oxidation. Finally, the addition of RBE did not show any overall effect, even punctually increasing the protein oxidations for GL/CH in low doses at day 1, but with similar values to control meat (*p* > 0.05). Despite the scarcity of literature about the implications of biofilms in protein oxidation of meat, due to the complexity of the mechanisms involved in protein oxidation [47], some studies reveal the protective effect of chitosan and gelatin coats against lipid and protein oxidations in fresh meat [46], especially when natural extracts were added [14]. However, similarly to our study, variable effects linked to both biopolymers are found. Thus, Antoniewski et al. [4] reported no effect of gelatin/chitosan coats in lipid oxidation for pork meat, whereas Guo et al. [48] observed that pork meat sprayed with chitosan increased the lipid oxidations, and Cardoso et al. [49] exposed as chitosan and gelatin-based coats also increased the TBA-RS values. With all, it would be interesting to point out that chitosan films should be formulated with the addition of some antioxidant additive to mitigate the pro-oxidant effect on meat. Though the addition of RBE did not demonstrate a general antioxidant effect on pork loin in the current assay, except for CH at day 9, previous studies have described the antioxidant effect of direct application in meat and other lipid matrices [20,50]. These results are in line with the previous bibliography, that shows that the addition of active extracts did not always prevent the oxidation preservation of meat proportionally to the extract dose for chitosan or gelatin films [17,51,52,53], included the RBE [54]. On the other hand, the absence of RBE migrations in simulant D1 for both films could explain the lack of antioxidant effects of RBE on loin pork meat, due to our meat samples, with 1.61% fat content, have a hydrophilic character comparable to the D1 simulant. This fact, and the storage at 4 °C, could limit the release of active compounds from the film to the meat matrix. Moreover, the addition of RBE can increase the permeability of films to oxygen, water and other oxidizing agents, consequently counteracting the antioxidant activity of γ-oryzanol released into the meat matrix. All these effects could explain the ineffectiveness of the addition of RBE to the GL/CH and CH films on the oxidative status of the meat throughout the storage period.

#### 2.2.3. Microbiological Analysis in Pork Loin

Considering the microbiological analysis in meat samples, the impact of films at different storage times is shown in Table 7 and Table 8, the results expressed as log CFU g^−1^.

In general, though for both films a preservative effect was observed, the CH films showed a significant advantage (*p* ≤ 0.05) in the control of mesophiles, psychrophiles, moulds and yeast, as well as total coliforms bacteria. Protection of meat with films was effective against aerobic mesophiles throughout the entire assay for GL/CH and CH, but differences between formulations were found. At day 1, for GL/CH only L-RBE showed lower mesophile loads than the control (*p* ≤ 0.01), and all CH formulations reduced the counts (*p* ≤ 0.001), especially the L-RBE and H-RBE batches. At day 5, all films helped to control the mesophiles (*p* ≤ 0.001), resulting in the most protective no-RBE for GL/CH and L-RBE and H-RBE for CH. At the end of the storage, GL/CH and CH significantly reduced (*p* ≤ 0.001) the counts of mesophiles, with no differences due to the RBE addition (*p* > 0.05). Comparing the two types of films, the highest effectivity was linked at day 1 to CH with L-RBE and H-RBE dose (*p* ≤ 0.01), at day 5 GL/CH L-RBE and CH L-RBE and H-RBE, and at day 9 for all CH formulations. The addition of RBE only enhanced the antimicrobial effect for CH at days 1 and 5, being indifferent to the added dose (*p* > 0.05). Thus, at the end of the storage period, the addition of RBE did not increase the antimicrobial effectiveness against mesophiles for any film. Regarding the psychrophile loads, the GL/CH films were not effective at days 1 and 5 (*p* > 0.05), whereas CH reduced the counts (*p* ≤ 0.001) in more than 2.4 units with all formulations at day 1, and at day 5 significantly decreased the counts in 4.7 units for no-RBE and more than 5.7 with the addition of RBE (*p* ≤ 0.001). At day 9, for GL/CH only the formulations with RBE showed an antimicrobial effect (*p* ≤ 0.001), whereas all CH formulations reduced the loads under the limit of detection (*p* ≤ 0.001). With all, CH resulted more effective (*p* ≤ 0.01) against psychrophiles than GL/CH, but for L-RBE at day 1 and no-RBE at day 5. Finally, the addition of RBE slightly improved the control of psychrophiles for CH at day 5 and for GL/CH at day 9. For moulds and yeasts, both films were effective in controlling their growth during all storage, especially with CH. At day 1, GL/CH only resulted effective for L-RBE (*p* ≤ 0.05), whereas for CH all the formulations significantly (*p* ≤ 0.001) decreased the moulds and yeasts counts. At day 5, GL/CH films did not show any effect (*p* > 0.05), whereas the CH films kept effective (*p* ≤ 0.001) with similar counts in all the formulations. At the end of the storage, both biopolymers decreased (*p* ≤ 0.001) the loads with all formulations, reaching the lowest counts in GL/CH films for no-RBE, and in CH for H-RBE. Against moulds and yeasts, CH was more protective than GL/CH throughout the assay (*p* ≤ 0.01), except in the no-RBE formulation at day 9. Overall, the inclusion of RBE had no effect, but H-RBE for CH at day 9 which improved the film effectiveness, reaching loads of 1.33 log CFU g^−1^.

Although both biopolymers helped to control the total coliform populations in fresh pork meat, the CH biopolymers were highly effective, keeping the counts under the detection limits in all the samples. The GL/CH films had no effect at day 1 (*p* > 0.05) and at day 5 helped to control the total coliform growths, with similar loads for all formulations. At day 9, only the no-RBE resulted effective for GL/CH. The inclusion of RBE in the GL/CH biopolymers did not affect the coliform control, even increasing the loads at day 9 in GL/CH films, which is considered an important disadvantage. The presence of *E. coli* and *S. aureus* was sporadic, detected only in some samples with loads too low to be considered significant. *E. coli* only appeared in some control samples at day 1. For *S. aureus*, an analogous situation occurred, being detected only in some control and GL/CH or CH film samples, and under the limit of detection (100 CFU g^−1^) in the majority of batches.

According to Codex Alimentarius, the control meat samples were within their shelf-life only until day 1, with mesophiles and psychrophiles counts above 6 log CFU g^−1^ from day 5. However, GL/CH and CH films effectively protected the fresh pork meat and kept it within the shelf-life until day 9, except for psychrophiles with GL/CH no-RBE formulation at day 9. None of the samples showed the presence of *Salmonella* spp., *Listeria monocytogenes* or *Clostridium perfringens*, and *E. coli* loads were within the limits established by the European regulation (Commission Regulation (EC) No 2073/2005). Considering the previous bibliography and the results of the antimicrobial in vitro activity of films in the present study (Table 3), the better antimicrobial behavior observed for CH films versus GL/CH was expected. In this sense, according to the antimicrobial activity observed in vitro for chitosan films in the present study against *E. coli* and *L. innocua,* the effectiveness as an antimicrobial agent in meat products has been widely demonstrated [46,51]. However, in contrast to the synergistic effect of chitosan with cellulose [55] or gelatin [56] in meat preservation described in the previous literature, in the present study the formulation with GL/CH decreased the antimicrobial potential of pure CH films, as shown in vitro in the present study, although a protective effect on meat was kept for GL/CH formulations. On the other hand, despite the enhancement of the bactericide activity in vitro with the addition of RBE to CH films (Table 3), the RBE did not improve the antimicrobial activity for meat preservation, with a variable trend depending on the film and the microorganism. This differential behavior has been also found in the previous literature. Thus, for chitosan-based films, the addition of natural extracts or essential oils improves the microbial control in meat [14,42,55]. However, in concordance with the present study, this tendency did not always occur. In this sense, some of the literature reports that the addition of rosemary essential oil to chitosan did not improve the antimicrobial effect in meat of chitosan films [52]; that the addition of tea extract to chitosan coatings did not enhance the effectiveness to control psychotropic and mesophiles load for pork chops [51], resulting indifferent the addition of active extracts; or that, for pork preservation with chitosan and gelatin/chitosan coatings, the incorporation of grape seed extracts in coats resulted indifferent [56]. On the other hand, for active chitosan, the effect of the extract dose on the microbial control for refrigerated pork meat depends on the microorganism [57]. In particular for RBE, in pork burgers with RBE spread on the meat surface and packaged with conventional film, no significant effects on mesophilic, psychrophiles and lactic acid aerobic bacteria at day 1 were found, although after 21 days of conservation, the burgers treated with hydrostatic high pressure and with RBE decreased the counts of lactic acid bacteria [20]. In the same way, Martillanes et al. [22], for Iberian ham packaged with conventional films and stored for 180 days, the addition of RBE only was effective against moulds and yeasts. Oppositely, Cabeza de Vaca et al. [13] did not find any effective control of microorganisms for fresh pork loin meat preserved with active PLA/PHB films with RBE. This differential behavior could be due to properties of active biopolymers, since the PLA, PHB and RBE are lipophilic compounds. Hence, the RBE in PLA/PHB films was retained chemically, whereas in chitosan and gelatin films, with a hydrophilic character, the retention resulted by the physical entrapment of the extracts in the porous of the net. Due to the low-fat content of the pork loin, in contrast with the hydrophobic nature of RBE, the migration of RBE in PLA or PHB films would unlikely occur within the meat matrix, as Cabeza de Vaca et al. [11] observed. However, higher mobility of RBE toward fatty meat products could be expected in the case of lipophilic films, with a positive antimicrobial effect of the RBE addition in food, such as Martillanes et al. [13,22,58] showed for Iberian ham packaged with conventional active films with RBE. Conversely, with chitosan and gelatine, which have a hydrophilic character, the contact of the films with the meat water can cause the relaxation and opening of the biopolymer net [59]. This could facilitate the contact of RBE with the meat surface and leads to an antimicrobial effect. However, Requena et al. [3] demonstrated that the antimicrobial activity of the extracts and the migration of the active compounds to the matrix do not always result in a reduction of microbial loads in food.

## 3. Conclusions

The active films developed from gelatin and chitosan biopolymers (GL/CH and CH) with rice bran extract (RBE) displayed interesting properties in vitro. The GL/CH films showed a higher release of γ-oryzanol and higher antioxidant activity than CH films. However, CH films exhibited a remarkable antimicrobial effect, which the GL/CH did not show.

When tested on pork meat, both GL/CH and CH films reduced the paleness of meat during storage, which is a positive outcome. The biofilms of CH notably increased the meat redness, potentially altering its overall appearance and leading to consumer rejection. Moreover, CH biopolymer promoted lipid oxidation during refrigerated storage, suggesting that the addition of an alternative antioxidant can be recommended for this type of film. Despite this, both films showed significant antimicrobial effects, particularly the CH one, which is highly interesting as they can significantly increase the shelf-life and the safety of meat products.

Nevertheless, future research should assess whether changes in color and lipid oxidation in fresh meat are noticeable by consumers and could lead to the rejection of fresh meat products. In such cases, the addition of RBE into active films would not be advantageous. Future studies should focus on a deeper analysis of the internal structure of the developed films and their interaction with the incorporated bioactive compounds to better understand their potential applications in food matrices. This understanding could enable a more accurate correlation between in vitro and in vivo results of the active biofilms in meat food applications.

## 4. Materials and Methods

### 4.1. Acquisition of Rice Bran Extract

Organic Bran rice was acquired from a local market and stored in refrigeration (4 °C) and darkness. The chemical composition according to the supplier was: moisture 10.0%, protein 11.8%, carbohydrate 19.0%, sugars 3.9%, total fat 19.4%, saturated fat 3.9%, dietary fiber 28.8% and salt 0.04%.

The rice bran extract (RBE) was obtained by extraction with ethanol (100%) in a ratio bran:ethanol of 1:10 (*w*/*v*), heating in a bath with shaking (Selecta, Unitronic OR model, Barcelona, Spain) at 60 °C for 97 min. The analysis of the content of γ-oryzanol by HPLC (Agilent Technologies, Waldbronn, Germany) was carried out according to Martillanes et al. [19]. Specifically, an 1100 Series HPLC system (Agilent Technologies, Waldbronn, Germany), equipped with a quaternary pump, a degasser, an auto-sampler and a diode array detector (DAD), was used to separate the analytes. The analytical column employed was a C-18 Gemini-NX (Phenomenex, Torrance, CA, USA, 3 μm, 4.6 mm × 150 mm) column, maintained at 40 °C by a thermostat and the chromatographic data processing was performed using HP ChemStation software (Rev.B.04.01., Agilent Technologies, CA, USA). The total antioxidant activity by the ABTS method [60] and the antibacterial activity by the bacteriostatic method [13] were also determined for RBE.

### 4.2. Preparation and Characterization of Films

#### 4.2.1. Film Formation

For the present study, commercial fish gelatin powdered (250 bloom) (GL) (Sosa, Barcelon, Spain) and chitosan of high molecular weight (CH) (Sigma Aldrich, Madrid, Spain, 419419), were used as biopolymers. The films formulations were as follows: the GL/CH solution was prepared in water with 1% (*w*/*v*) of Chitosan, 0.4% (*v*/*v*) acid lactic, 3% (*w*/*v*) of gelatin powder and 0.4% (*v*/*v*) of glycerol as a plasticizer, and homogenised by stirring for 30 min with temperature (50 °C) (Selecta, Agimatic-N model, Barcelona, Spain); the CH solution was obtained dissolving 2% of chitosan (*w*/*v*) in a 1% (*v*/*v*) aqueous lactic acid and with 1% (*v*/*v*) of glycerol, in stirring for 30 min with temperature (50 °C). Finally, 0% (no RBE), 0.3% (low dose: L-RBE) or 0.5% (high dose: H-RBE) of RBE was added (*w*/*v*) to each biopolymer solution. Thus, six formulations of films were obtained: GL/CH no RBE, GL/CH L-RBE, GL/CH H-RBE, CH no RBE, CH L-RBE and CH H-RBE (Supplementary Material). Film solutions (20 g) were poured into polystyrene Ø 90 mm Petri dishes and dried at room temperature in the dark for 48–72 h until film formation.

#### 4.2.2. Migrations, Antioxidant Capacity and Antimicrobial Characterization of Films

The RBE migrations from the biofilms were measured as total γ-oryzanol released for simulants with intermediate (D1) and low polarities (D2), according to migration methods (European Commission regulation No. 10/2011). Two standard food simulants were utilised: 50% ethanol (*v*/*v*) as D1, and 95% ethanol (*v*/*v*) as D2. The analysis was carried out according to Cabeza de Vaca et al. [13] by direct immersion of a piece of each biopolymer (10 × 15 mm^2^) in the simulant dilutions (10 mL). The releases were measured at different times from 2 to 240 h with agitation in a shaker (Comecta, shaker D-2102 model, Barcelona, Spain). The migrated RBE was determined as total γ-oryzanol released by HPLC, according to Martillanes et al. [19], similarly to the determination of the analysis of the content of γ-oryzanol in the extraction of RBE (Section 2.1). Results of migrations were expressed as µg γ-oryzanol cm^−2^ released.

Total antioxidant activity (TAA) of films was determined spectrophotometrically by ABTS radical scavenging method for lipophilic samples [60] at 2, 6 and 24 h, and using a plate reader (Tecan, spark multimode microplate reader model, Grödig, Austria) according to Cabeza de Vaca et al. [13]. Trolox was used as the standard. Results were expressed as µg of equivalent Trolox mm^−2^.

The antimicrobial inhibitory capacity of films was determined against *Escherichia coli* (CECT 45) and *Listeria innocua* (CECT 910), according to bacteriostatic activity method defined by Cabeza de Vaca et al. [13]. Thus, bacterial cultures stored at −80 °C were regenerated with Mueller–Hinton broth media. The incubation was carried out at 37 °C for 20 h under shaker (Comecta, shaker D-2102 model, Barcelona, Spain). The fresh cultures were diluted into Mueller–Hinton broth media to obtain a load of 10^6^ CFU (colony forming units) mL^−1^. Afterward, 100 µL of the cultures were transferred to 10 mL of Mueller–Hinton broth media (concentration in tube 10^4^ CFU) and the film pieces (6 mm of diameter) of each formulation were placed in the inoculated tubes, with no film added for the control. Subsequently, the samples were incubated at 37 ◦C for 24 h. After the incubation, the final microbial growth was quantified (log CFU mL^−1^) by culturing on Petri dishes with standard plate count agar (PCA, Merck, Darmstadt, Germany), incubated for 72 h at 30 °C in aerobiosis. These three analyses above were carried out in triplicate for each film formulation.

### 4.3. Experimental Design and Packaging of Pork Loin

The fresh pork loins were acquired from a local butcher. The loins were sliced into 1 cm steaks. The proximal composition of meat was: pH 5.6, Moisture 73.2%, protein 24.1%, and total fat 1.6%. In the experimental design, steaks with no film were used as control. Each one of the steaks was covered with one of the six biopolymer formulations on both surfaces. All steaks, both with films and the control, were individually vacuum packaged (−0.8 bar) using a Henkovac Proeco equipment (Henkovac International, Hertogenbosch, Netherlands) in 10 × 10 cm^2^ polyamide polyethylene (20/100) bags of 120 µm thickness (OptiDure™ ODA7005, oxygen permeability: 10 cm^3^ m^−2^, 24 h^−1^, and 0% relative humidity; Cryovac, Madrid, Spain) and stored for 1, 5 or 9 days at 4 °C in darkness. At the end of the storage, the films were removed for each steak and the microbiological loads and instrumental color were immediately determined. Finally, the remaining portion of each fillet was frozen at −80 °C until the analysis of lipid and protein oxidations. A set of five steaks was analyzed for each combination of film treatment and storage time. Therefore, a total of 120 loin steaks were evaluated (biopolymers (2) × formulations (4) × replicates (5) × storage time (3)).

#### 4.3.1. Instrumental Colour Parameters

Colour was measured by duplicated on both surfaces of the steak samples with a Konica Minolta CM-5 Spectrophotometer (Konica Minolta, Tokyo, Japan). The reflectance was measured, and an illuminant D65, a viewing angle of 10° and a 30 mm diameter of the aperture were used for the measures.

The colour parameters were reported in the CIELAB scale with the parameters L* (lightness), a* (redness) and b* (yellowness). Chroma (saturation) and Hue (angle tone) were determined throughout the equations: Chroma = √(a*^2^ + b*^2^); Hue = atang(b*/a*).

Finally, two types of comparison of total color variations (ΔE) were also determined. ΔE_initial_control_ quantified the color deviations linked to film and time simultaneously for each sample, with respect to the original control meat at day 1. ΔE_storage_ determines the color evolution of a sample due to the conservation time within each film batch, concerning the corresponding sample at day 1. Both ΔE were calculated throughout the equations:

$${\Delta E}_{initial\_control}=\sqrt{{\left(L^{*}-L_{initial\_control}^{*}\right)}^{2}+{\left(a^{*}-a_{initial\_control}^{*}\right)}^{2}+{\left(b^{*}-b_{initial\_control}^{*}\right)}^{2}}$$

$${\Delta E}_{storage}=\sqrt{{\left(L^{*}-L_{D1}^{*}\right)}^{2}+{\left(a^{*}-a_{D1}^{*}\right)}^{2}+{\left(b^{*}-b_{D1}^{*}\right)}^{2}}$$

#### 4.3.2. Oxidative Status

The thiobarbituric reactive substances (TBA-RS) method [61] was carried out to determine the meat lipid oxidations, and malonaldehyde (MDA) was used as standard. Results were expressed as mg MDA kg^−1^ meat.

Protein oxidation was determined spectrophotometrically following the Oliver et al. method [62], which is based on the measure of the ratio of carbonyls/protein. The carbonyl concentrations were calculated by measuring the absorbance of 2,4-dinitrophenylhydrazine (DNPH) linked to carbonyl groups of meat protein at 370 nm, with an absorption coefficient of 21.0 mM^−1^ cm^−1^ for protein hydrazones. Protein concentration was determined spectrophotometrically at 280 nm with bovine serum albumin (BSA) as standard. Results were expressed as nmol of carbonyls mg^−1^ protein.

#### 4.3.3. Microbiological Analysis

To evaluate the microbial status in meat, loads of mesophiles, psychrophiles, moulds and yeast, total coliforms bacteria, *Escherichia coli* and *Staphylococcus aureus* were measured at days 1, 5 and 9 of refrigeration. The presence of *Salmonella* spp. and *Listeria monocytogenes* was also determined (Commission Regulation (EC) No 2073/2005).

Thus, under aseptic conditions, a portion of each meat sample (10 g) was homogenized and blended (Stomacher^®^ 400 Circulator) with peptone water (Merck, 1.07043) (90 mL). Subsequently, serial decimal dilutions were made in sterile peptone water. 1 mL of the appropriate sample dilutions was poured or 100 µL spread onto total count and selective agar plates. The cultures were carried out in adequate agar media and conditions according to ISO standards. Total mesophiles and psychrophiles were performed according to ISO 4833-1:2013 [63], with a standard Plate Count Agar (PCA) (Merck, Darmstadt, Germany), and incubated at 30 °C for 72 h and 6.5 °C for 7 days, respectively. Total moulds and yeasts were determined by count in YGC Agar (Merck, Darmstadt, Germany), and the incubation was carried out at 25 °C for 6 days, according to ISO 21527-2:2008 [64]. For the total coliforms [65] and *Escherichia coli* [66], Chromocult Agar media was used (Merck, 1.10426), and the incubation was carried out at 37 °C for 24–48 h. *Staphylococcus aureus* loads were determined according to ISO 6888 1/2:2021 [67], after incubation at 37 °C for 24–48 h in Baid Parker Agar (Merck, Darmstadt, Germany, 1.05406). The presence or absence of *Salmonella* spp. and *Listeria monocytogenes* was determined according to ISO 6579-1:2017 [68] and ISO 11290-1:2017 [69], respectively, from 12.5 g of fresh meat.

All Results were expressed as Log10 CFU (colony forming units)/g. The detection limit of each microorganism was 10 CFU/g, but for *S. aureus*, it was 100 CFU/g.

### 4.4. Statistical Analysis

The results are shown as mean ± standard deviation. The interactions between biopolymers, RBE concentration and storage time factors were determined by the analysis of the variance of one, two and three ways. The effects of biopolymer type or film formulation were analyzed with a one-way analysis of variance (ANOVA). For the film formulation, when ANOVA showed significant differences. In the case of significant differences for more than two groups, a Tukey’s test was applied to compare their mean values. The software used for these statistical analyses was SSPS(IBM Corp. Released 2020. IBM SPSS Statistics for Windows, Version 27.0. IBM Corp, Armonk, NY, USA). A significant value of *p* ≤ 0.05 was established.

## Figures and Tables

**Table 1:** Evolution of RBE release in vitro (γ-oryzanol µg cm^−^^2^) in simulant D2 (95% ethanol) of active GL/CH and CH active films enriched with RBE.

	GL/CH		CH		Significance		
Migration Time	L-RBE	H-RBE	L-RBE	H-RBE	Film	RBE	Film × RBE
2 h	8.69 ± 2.38 by	16.04 ± 2.84 az	4.70 ± 1.42 bc	2.26 ± 0.33 cyz	***	ns	**
6 h	19.74 ± 3.18 ax	18.08 ± 1.80 ayz	5.41 ± 1.61 b	1.69 ± 0.73 cz	***	ns	ns
12 h	13.22 ± 3.28 axy	16.25 ± 2.93 az	5.22 ± 1.07 b	2.33 ± 0.25 byz	***	ns	ns
24 h	19.93 ± 5.63 ax	21.13 ± 2.12 ayz	5.38 ± 0.94 b	2.89 ± 0.37 bxyz	***	ns	ns
48 h	16.69 ± 1.43 bx	23.75 ± 3.52 axyz	5.91 ± 1.99 c	4.06 ± 0.14 cxy	***	ns	**
72 h	18.45 ± 3.15 bx	25.46 ± 2.50 axy	8.46 ± 2.00 b	4.09 ± 1.45 cxy	***	ns	**
144 h	18.64 ± 2.13 bx	30.82 ± 4.61 ax	8.12 ± 2.2 b	4.07 ± 1.28 cxy	***	*	**
240 h	16.4 ± 1.74 bx	25.35 ± 2.51 axy	7.05 ± 0.81 b	4.86 ± 0.67 cx	***	**	***
Significance	**	**	ns	**			

GL/CH: gelatin/chitosan. CH: chitosan. L-RBE: 0.3% of rice extract added to film. H-RBE: 0.5% of rice extract added to film. Results are expressed as MEAN ± SD. a, b, c: different letters in the same row indicate statistical differences. x, y, z: different letters in the same column indicate statistical differences. Tukey’s test significance levels: ns *p* > 0.05; * *p* ≤ 0.05; ** *p* ≤ 0.01; *** *p* ≤ 0.001.

**Table 2:** ABTS total antioxidant activity (ug Trolox equivalent cm^−2^) of active GL/CH and CH films.

	GL/CH			CH			Significance		
TAA Time	No RBE	L-RBE	H-RBE	No RBE	L-RBE	H-RBE	Film	RBE	Film × RBE
2 h	19.19 ± 0.97 ay	18.13 ± 1.71 a	17.95 ± 1.27 az	6.46 ± 0.46 b	3.37 ± 0.29 cy	3.57 ± 1.49 c	***	*	ns
6 h	20.33 ± 0.94 by	21.5 ± 0.53 ab	22.37 ± 0.53 ay	6.60 ± 0.06 c	1.60 ± 0.36 exy	2.61 ± 0.24 d	***	***	***
24 h	23.34 ± 0.70 bx	23.44 ± 4.67 b	29.11 ± 0.12 ax	7.27 ± 0.47 c	3.81 ± 1.27 dx	4.01 ± 1.32 d	***	ns	*
Sig.	**	ns	***	ns	*	ns			

TAA: total antioxidant activity. GL/CH: gelatin/chitosan. CH: chitosan. L-RBE: 0.3% of rice extract added to film. H-RBE: 0.5% of rice extract added to film. Results are expressed as MEAN ± SD. a, b, c, d, e: different letters in the same row indicate statistical differences. x, y, z: different letters in the same column indicate statistical differences. Tukey’s test significance levels: ns *p* > 0.05; * *p* ≤ 0.05; ** *p* ≤ 0.01; *** *p* ≤ 0.001.

**Table 3:** Bacteriostatic antimicrobial activity in vitro of CH films (log CFU mL^−1^).

	Control	No RBE	L-RBE	H-RBE	Significance
*E. coli*					
0 h	5.79 ± 0.00 z	5.79 ± 0.00 x	5.79 ± 0.00 w	5.79 ± 0.00 x	ns
24 h	8.80 ± 0.01 ay	2.87 ± 0.01 cz	3.70 ± 0.21 bx	2.86 ± 0.03 cy	***
48 h	9.43 ± 0.11 ax	4.56 ± 0.06 by	3.24 ± 0.15 cy	2.63 ± 0.12 dy	***
72 h	9.86 ± 0.06 aw	5.77 ± 0.06 bx	2.87 ± 0.04 cz	2.18 ± 0.06 dz	***
Significance	***	***	***	***	
*L. innocua*					
0 h	5.72 ± 0.00 z	5.72 ± 0.00 x	5.72 ± 0.00 x	5.72 ± 0.00 x	ns
24 h	9.47 ± 0.34 ay	5.92 ± 0.08 bx	5.76 ± 0.22 bx	3.87 ± 0.05 cy	***
48 h	10.42 ± 0.12 ax	3.54 ± 0.12 bz	3.24 ± 0.21 by	2.54 ± 0.12 cz	***
72 h	10.59 ± 0.24 ax	4.53 ± 0.09 by	2.85 ± 0.12 cy	2.53 ± 0.09 cz	***
Significance	***	***	***	***	

CH: chitosan. Control: cell cultures without film. No-RBE: no rice bran extract added to film. L-RBE: 0.3% of rice extract added to film. H-RBE: 0.5% of rice extract added to film. Results are expressed as MEAN ± *SD*. a, b, c, d: different letters in the same row indicate statistical differences. w, x, y, z: different letters in the same column indicate statistical differences. Tukey’s test significance levels: ns *p* > 0.05; *** *p* ≤ 0.001.

**Table 4:** Interaction between effects of the three studied parameters: film type, RBE concentration and storage time.

	P1	P2	P3	P1 × P2	P1 × P3	P2 × P3	P1 × P2 × P3
CIE L*	**	ns	*	ns	***	ns	*
CIE a*	***	ns	ns	ns	ns	ns	ns
CIE b*	***	*	**	**	ns	*	*
Chroma	***	***	**	***	ns	*	ns
HUE	***	***	ns	***	ns	ns	ns
ΔE_initial_control_	ns	ns	**	***	*	**	ns
ΔE_storage_	ns	**	***	**	***	ns	ns
TBA-RS	***	ns	***	**	***	**	ns
Protein oxidation	*	ns	*	ns	ns	ns	*
Mesophiles	***	**	***	***	***	ns	**
Psychrophiles	***	ns	***	ns	***	ns	ns
Moulds and Yeasts	***	ns	***	**	*	***	***
Coliforms	***	***	***	***	***	***	***
*E. coli*	***	ns	ns	ns	*	ns	ns
*S. aureus*	*	ns	*	ns	ns	ns	ns

P1: film (no film, GL/CH and CH). P2: rice bran extract concentration added (without RBE, 0.3% RBE and 0.5% RBE). P3: storage time (day 1, day 5 and day 9). ANOVA test significance levels: ns *p* > 0.05; * *p* ≤ 0.05; ** *p* ≤ 0.01; *** *p* ≤ 0.001.

**Table 5:** Instrumental color, lipid oxidation (mg MDA kg^−1^ meat) and protein oxidation (nmol of carbonyls mg^−1^ protein) of pork loin steaks along the conservation period for each GL/CH and CH film treatment.

	Control	No RBE	L-RBE	H-RBE	Significance
CIE L* day 1					
GL/CH	54.83 ± 3.37	53.20 ± 1.54	52.86 ± 1.29	53.50 ± 2.09	ns
CH	53.56 ± 0.82	54.09 ± 2.24	55.4 ± 1.74	53.29 ± 0.96	ns
Significance	ns	ns	*	ns	
CIE L* day 5					
GL/CH	57.09 ± 2.41	55.11 ± 0.50	55.13 ± 0.96	54.64 ± 0.94	ns
CH	55.60 ± 3.32 ab	56.6 ± 2 a	51.4 ± 1.5 b	53.73 ± 3.08 ab	*
Significance	ns	ns	**	ns	
CIE L* day 9					
GL/CH	59.19 ± 1.90 a	56.24 ± 0.73 b	55.68 ± 0.90 b	55.95 ± 1.06 b	**
CH	57.05 ± 1.79 a	53.21 ± 3.16 ab	51.89 ± 1.73 b	53.71 ± 1.67 ab	*
Significance	ns	ns	**	*	
CIE a* day 1					
GL/CH	2.78 ± 1.70	2.43 ± 0.78	2.53 ± 0.38	2.29 ± 0.78	ns
CH	2.39 ± 0.53 b	4.14 ± 1.19 a	3.76 ± 0.38 a	4.17 ± 0.62 a	**
Significance	ns	*	**	**	
CIE a* day 5					
GL/CH	2.08 ± 1.58	2.74 ± 0.62	2.45 ± 0.34	2.36 ± 0.43	ns
CH	2.64 ± 1.42	4.10 ± 0.85	4.04 ± 1.24	4.19 ± 0.65	ns
Significance	ns	*	*	**	
CIE a* day 9					
GL/CH	1.82 ± 1.03	2.08 ± 0.59	2.12 ± 0.44	1.46 ± 0.42	ns
CH	1.65 ± 0.86 b	4.21 ± 0.59 a	4.23 ± 0.90 a	4.53 ± 0.69 a	***
Significance	ns	***	**	***	
CIE b* day 1					
GL/CH	9.15 ± 0.48 a	7.48 ± 0.82 b	7.94 ± 0.82 ab	8.16 ± 1.20 ab	*
CH	9.12 ± 0.56	9.16 ± 1.05	9.54 ± 0.73	8.99 ± 0.41	ns
Significance	ns	*	*	ns	
CIE b* day 5					
GL/CH	9.23 ± 0.33 a	7.28 ± 0.38 b	7.51 ± 0.33 b	7.58 ± 0.24 b	***
CH	9.97 ± 0.51 a	9.64 ± 1.16 a	6.90 ± 1.00 b	8.21 ± 1.56 ab	**
Significance	*	**	ns	ns	
CIE b* day 9					
GL/CH	9.30 ± 0.51 a	7.25 ± 0.46 b	7.08 ± 0.43 b	7.14 ± 0.26 b	***
CH	9.89 ± 0.40 a	8.61 ± 1.34 ab	7.01 ± 0.90 b	8.42 ± 1.28 ab	**
Significance	ns	ns	ns	ns	

GL/CH: gelatin/chitosan. CH: chitosan. Control: meat without film. No RBE: no RBE added film. L-RBE: 0.3% RBE. H-REB: 0.5% RBE. Results are expressed as MEAN ± SD. a, b: Different letters in the same row indicate statistical differences. Tuckey’s test significance levels: ns *p* > 0.05; * *p* ≤ 0.05; ** *p* ≤ 0.01; *** *p* ≤ 0.001.

**Table 6:** Lipid oxidation (mg MDA kg^−1^ meat) and protein oxidation (nmol of carbonyls mg^−1^ protein) of pork loin steaks along the conservation period for each GL/CH and CH film treatment.

	Control	No RBE	L-RBE	H-RBE	Significance
**TBA-RS day 1**					
GL/CH	0.18 ± 0.08	0.24 ± 0.07	0.19 ± 0.04	0.24 ± 0.04	ns
CH	0.13 ± 0.03 b	0.35 ± 0.20 a	0.31 ± 0.11 ab	0.32 ± 0.10 ab	*
Significance	ns	ns	*	ns	
TBA-RS day 5					
GL/CH	0.18 ± 0.06	0.19 ± 0.08	0.30 ± 0.10	0.31 ± 0.08	ns
CH	0.12 ± 0.03 b	0.38 ± 0.10 a	0.37 ± 0.07 a	0.42 ± 0.13 a	***
Significance	ns	*	ns	ns	
TBA-RS day 9					
GL/CH	0.22 ± 0.04	0.36 ± 0.13	0.38 ± 0.05	0.34 ± 0.16	ns
CH	0.16 ± 0.03 c	0.79 ± 0.07 a	0.53 ± 0.14 b	0.43 ± 0.19 b	***
Significance	*	***	ns	ns	
**Protein oxidation day 1**					
GL/CH	1.06 ± 0.14 ab	0.94 ± 0.11 b	1.17 ± 0.15 a	0.91 ± 0.05 b	*
CH	1.09 ± 0.11	1.02 ± 0.11	0.98 ± 0.05	1.12 ± 0.18	ns
Significance	ns	ns	*	*	
Protein oxidation day 5					
GL/CH	0.99 ± 0.21	0.97 ± 0.21	0.95 ± 0.18	1.00 ± 0.38	ns
CH	1.33 ± 0.20 a	0.97 ± 0.04 b	1.18 ± 0.17 ab	1.16 ± 0.15 ab	*
Significance	*	ns	ns	ns	
Protein oxidation day 9					
GL/CH	0.99 ± 0.21	0.97 ± 0.21	0.95 ± 0.18	1.00 ± 0.38	ns
CH	1.33 ± 0.20 a	0.97 ± 0.04 b	1.18 ± 0.17 ab	1.16 ± 0.15 ab	**
Significance	ns	ns	*	ns	

GL/CH: gelatin/chitosan. CH: chitosan. Control: meat without film. No RBE: no RBE added film. L-RBE: 0.3% RBE. H-REB: 0.5% RBE. TBA-RS: thiobarbituric reactive substances. Results are expressed as MEAN ± SD. a, b, c: different letters in the same row indicate statistical differences. Tuckey’s test significance levels ns *p* > 0.05; * *p* ≤ 0.05; ** *p* ≤ 0.01; *** *p* ≤ 0.001.

**Table 7:** Microbiological changes (log CFU g^−1^) during storage of pork loin steaks for each GL/CH and CH film.

	Control	No RBE	L-RBE	H-RBE	Significance
	**Mesophiles**				
**Day 1**					
GL/CH	5.46 ± 0.74 a	4.46 ± 0.44 ab	4.03 ± 0.78 b	5.03 ± 0.42 ab	*
CH	5.68 ± 0.15 a	4.43 ± 0.28 b	3.44 ± 0.15 c	3.37 ± 0.27 c	***
Significance	ns	ns	ns	**	
Day 5					
GL/CH	4.58 ± 0.22 a	2.81 ± 0.19 d	3.43 ± 0.20 c	4.02 ± 0.17 b	***
CH	6.39 ± 0.15 a	4.42 ± 0.33 b	2.80 ± 0.62 c	3.11 ± 0.53 c	***
Significance	***	***	ns	**	
Day 9					
GL/CH	6.83 ± 0.15 a	5.53 ± 0.24 b	5.51 ± 0.42 b	5.58 ± 0.30 b	***
CH	7.77 ± 0.25 a	4.33 ± 0.23 b	4.31 ± 0.34 b	4.12 ± 0.27 b	***
Significance	***	***	**	***	
	**Psychrophiles**				
**Day 1**					
GL/CH	4.21 ± 0.11	4.04 ± 0.48	3.84 ± 0.47	4.51 ± 0.32	ns
CH	5.13 ± 0.33 a	2.68 ± 0.38 b	2.58 ± 0.32 b	2.56 ± 0.22 b	***
Significance	**	**	ns	***	
Day 5					
GL/CH	4.58 ± 0.34	3.83 ± 1.05	4.51 ± 0.28	4.23 ± 0.32	ns
CH	8.67 ± 0.15 a	3.93 ± 0.29 b	3.01 ± 0.59 c	2.67 ± 0.59 c	***
Significance	***	ns	**	**	
Day 9					
GL/CH	6.59 ± 0.28 a	6.35 ± 0.25 a	5.48 ± 0.33 b	5.70 ± 0.16 b	***
CH	7.57 ± 0.20 a	nd b	nd b	nd b	***
Significance	***	***	***	***	
	**Moulds and yeasts**				
**Day 1**					
GL/CH	3.94 ± 0.42 a	3.46 ± 0.26 ab	3.10 ± 0.44 b	3.88 ± 0.38 a	*
CH	3.56 ± 0.15 a	1.78 ± 0.15 b	1.87 ± 0.13 b	2.03 ± 0.51 b	***
Significance	ns	***	**	***	
Day 5					
GL/CH	3.14 ± 1.22	3.55 ± 0.47	3.36 ± 0.13	3.27 ± 0.17	ns
CH	3.60 ± 0.17 a	1.71 ± 0.36 b	1.43 ± 0.74 b	1.60 ± 0.37 b	***
Significance	ns	***	***	***	
Day 9					
GL/CH	4.86 ± 0.05 a	3.02 ± 0.31 c	3.94 ± 0.54 b	3.75 ± 0.47 b	***
CH	3.83 ± 0.17 a	2.79 ± 0.41 b	2.96 ± 0.10 b	1.33 ± 0.52 c	***
Significance	***	ns	**	***	

GL/CH: gelatin/chitosan. CH: chitosan. Control: meat without film. No RBE: no RBE added film. L-RBE: 0.3% RBE. H-REB: 0.5% RBE. Results are expressed as MEAN ± SD. nd means below the detection limit of the method: nd < 1 log CFU g^−1^. a, b, c, d: different letters in the same row indicate statistical differences. Tuckey’s test significance levels: ns *p* > 0.05; * *p* ≤ 0.05; ** *p* ≤ 0.01; *** *p* ≤ 0.001. Abbreviations: CFU, colony forming units.

**Table 8:** Microbiological changes (log CFU g^−1^) during storage of pork loin steaks for each GL/CH and CH film.

	Control	No RBE	L-RBE	H-RBE	Significance
	**Total coliforms**				
**Day 1**					
GL/CH	2.11 ± 0.37	2.01 ± 0.74	1.72 ± 0.72	1.27 ± 0.29	ns
CH	2.55 ± 0.39 a	nd b	nd b	nd b	***
Significance	ns	**	**	***	
Day 5					
GL/CH	2.95 ± 0.17 a	1.11 ± 0.28 b	1.89 ± 0.76 b	1.40 ± 0.61 b	***
CH	2.90 ± 0.06 a	nd b	nd b	nd b	***
Significance	ns	***	**	**	
Day 9					
GL/CH	3.58 ± 0.39 a	1.55 ± 0.38 b	3.61 ± 0.19 a	3.62 ± 0.29 a	***
CH	2.66 ± 0.28 a	nd b	nd b	nd b	***
Significance	**	***	***	***	
	***E. coli***				
**Day 1**					
GL/CH	nd	nd	nd	nd	ns
CH	1.30 ± 0.40	nd	nd	nd	ns
Significance	ns	ns	ns	ns	
Day 5					
GL/CH	nd	nd	nd	nd	ns
CH	nd	nd	nd	nd	ns
Significance	ns	ns	ns	ns	
Day 9					
GL/CH	nd	nd	nd	nd	ns
CH	nd	nd	nd	nd	ns
Significance	ns	ns	ns	ns	
	***S. aureus***				
**Day 1**					
GL/CH	ND	ND	ND	ND	ns
CH	2.07 ± 0.15	ND	2.00 ± 0.0	ND	ns
Significance	ns	ns	ns	ns	
Day 5					
GL/CH	ND	ND	ND	ND	ns
CH	2.09 ± 0.22	ND	ND	ND	ns
Significance	ns	ns	ns	ns	
Day 9					
GL/CH	2.21 ± 0.30	2.00 ± 0.00	2.00 ± 0.00	2.15 ± 0.35	ns
CH	2.21 ± 0.30	ND	ND	ND	ns
Significance	ns	ns	ns	ns	

GL/CH: gelatin/chitosan. CH: chitosan. Control: meat without film. No RBE: no RBE added film. L-RBE: 0.3% RBE. H-REB: 0.5% RBE. Results are expressed as MEAN ± SD. nd and ND means below the detection limit of the method: nd < 1 log CFU g^−1^; ND < 2 log CFU g^−1^. a, b: different letters in the same row indicate statistical differences. Tuckey’s test significance levels: ns *p* > 0.05; ** *p* ≤ 0.01; *** *p* ≤ 0.001. Abbreviations: CFU, colony forming units.

## Data Availability

The raw data supporting the conclusions of this article will be made available by the authors on request.

## Supplementary Materials

The following supporting information can be downloaded at: https://www.mdpi.com/article/10.3390/gels11050338/s1, Table S1. Formulations of edible film solutions. Table S2. Chroma and Hue and calculated color changes of pork loin steaks along the conservation period for each GL/CH and CH film.
