# Supplementary material for: Effects of Gelatin/Chitosan and Chitosan Active Films with Rice Bran Extract for the Preservation of Fresh Pork Meat
Source: Gels. 2025 Apr 30;11(5):338. doi: 10.3390/gels11050338 (PMC12111214; doi:10.3390/gels11050338)
Supplement: Supplementary file 1 [file gels-11-00338-s001.zip › gels-3610319-supplementary.pdf]

Supplementary Materials

Table S1. Formulations of edible film solutions.

Type of film	Formulation
Gelatine/chitosan no RBE	1% chitosan + 3% gelatine
Gelatine/chitosan RBE Low	1% chitosan + 3% gelatine + 0.3% RBE
Gelatine/chitosan RBE High	1% chitosan + 3% gelatine + 0.5% RBE
Chitosan no RBE	2% chitosan
Chitosan RBE Low	2% chitosan + 0.3% RBE
Chitosan RBE High	2% chitosan + 0.5% RBE

Table S2. Chroma and Hue and calculated color changes of pork loin steaks along the conservation period for each GL/CH and CH film.

	Control	No-RBE	L-RBE	H-RBE	Significance
**Chroma day 1**					
GL/CH	9.77±0.71 a	7.91±0.68 b	8.34±0.86 ab	8.5±1.29 ab	*
CH	9.43±0.51	10.1±1.19	10.27±0.65	9.88±0.46	ns
Significance	ns	**	**	ns	
Chroma day 5					
GL/CH	9.56±0.64 a	7.79±0.53 b	7.91±0.28 b	7.95±0.25 b	***
CH	10.39±0.49 a	10.5±1.30 b	8.02±1.45 b	9.28±1.37 ab	*
Significance	*	**	ns	ns	
Chroma day9					
GL/CH	9.52±0.57 a	7.61±0.59 b	7.4±0.41 b	7.29±0.31 b	***
CH	10.05±0.51	9.56±1.44	8.2±1.16	9.57±1.42	ns
Significance	ns	*	ns	**	
**Hue day1**					
GL/CH	73.35±12.25	71.87±6.41	72.31±2.07	74.58±4.46	ns
CH	75.55±3.81 a	65.99±6.2 b	68.46±2.68 ab	65.97±4.63 b	*
Significance	ns	ns	*	*	
Hue day5					
GL/CH	77.75±8.81	69.58±3.84	71.93±2.8	72.74±3.02	ns
CH	75.29±7.76 a	67.01±3.36 ab	60.37±5.22 b	62.53±6.54 b	**
Significance	ns	ns	**	*	
Hue day9					
GL/CH	79.02±5.85	73.18±4.39	73.39±3.59	78.48±3.07	ns
CH	80.68±4.43a	64.36±3.11 b	59.11±3.51 b	61.65±1.98 b	***
Significance	ns	**	***	***	
**ΔE Initial control day1**					
GL/CH	0.00±0.00 b	2.62±1.42 a	2.47±1.28 a	2.44±1.63 a	*
CH	0.00±0.00 b	2.83±1.29 a	2.63±1.5 a	1.97±0.81 a	*
Significance	ns	ns	ns	ns	
ΔE _E Control T0 day5					
GL/CH	3.11 ±1.82	2.02±0.41	1.93±0.29	1.85±0.40	ns
CH	3.24±2.52	3.82±1.83	3.85±1.3	3.45±1.6	ns
Significance	ns	ns	*	ns	
ΔE_E Control T0 day9					
GL/CH	4.59±1.88 a	2.64±0.27 b	2.5±0.46 b	2.84±0.25 ab	*
CH	3.79±1.68	3.47±1.35	3.66±1.07	2.93±0.73	ns
Significance	ns	ns	ns	ns	
**ΔE _ storage day 1**					
GL/CH	0.00±0.00	0.00±0.00	0.00±0.00	0.00±0.00	
CH	0.00±0.00	0.00±0.00	0.00±0.00	0.00±0.00	
Significance					
ΔE _ T0 day 5					
GL/CH	3.11±1.82	2.06±0.43	2.38±0.85	1.45±0.74	ns
CH	3.24±2.52	3.02±1.69	5.02±1.76	2.98±1.5	ns
Significance	ns	ns	*	ns	
ΔE_ T0 day 9					

GL/CH	4.59±1.88	3.19±0.64	3.06±0.74	2.93±0.61	ns
CH	3.79±1.68	2.87±1.85	4.56±1.75	1.92±1.04	ns
Significance	ns	ns	ns	ns	

GL/CH: gelatin/chitosan.CH: chitosan.Control: meat without film. No RBE: no RBE added film. L-RBE: 0.3% RBE. H-REB: 0.5% RBE. Results are expressed as MEAN ± SD. a, b: different letters in the same row indicates statistically differences. Tuckey´s test significance levels ns $p>0.05$; * $p \leq 0.05$; ** $p \leq 0.01$; *** $p \leq 0.001$.
